# Supplementary material for: Seminoma and Embryonal Carcinoma Footprints Identified by Analysis of Integrated Genome-Wide Epigenetic and Expression Profiles of Germ Cell Cancer Cell Lines
Source: PLoS One. 2014 Jun 2;9(6):e98330. doi: 10.1371/journal.pone.0098330 (PMC4041891; doi:10.1371/journal.pone.0098330)
Supplement: File S1 — ZIP file containing DMRforPairs output for significant regions. Please start from the html files. (ZIP) [file pone.0098330.s008.zip › figures/19102.pdf]

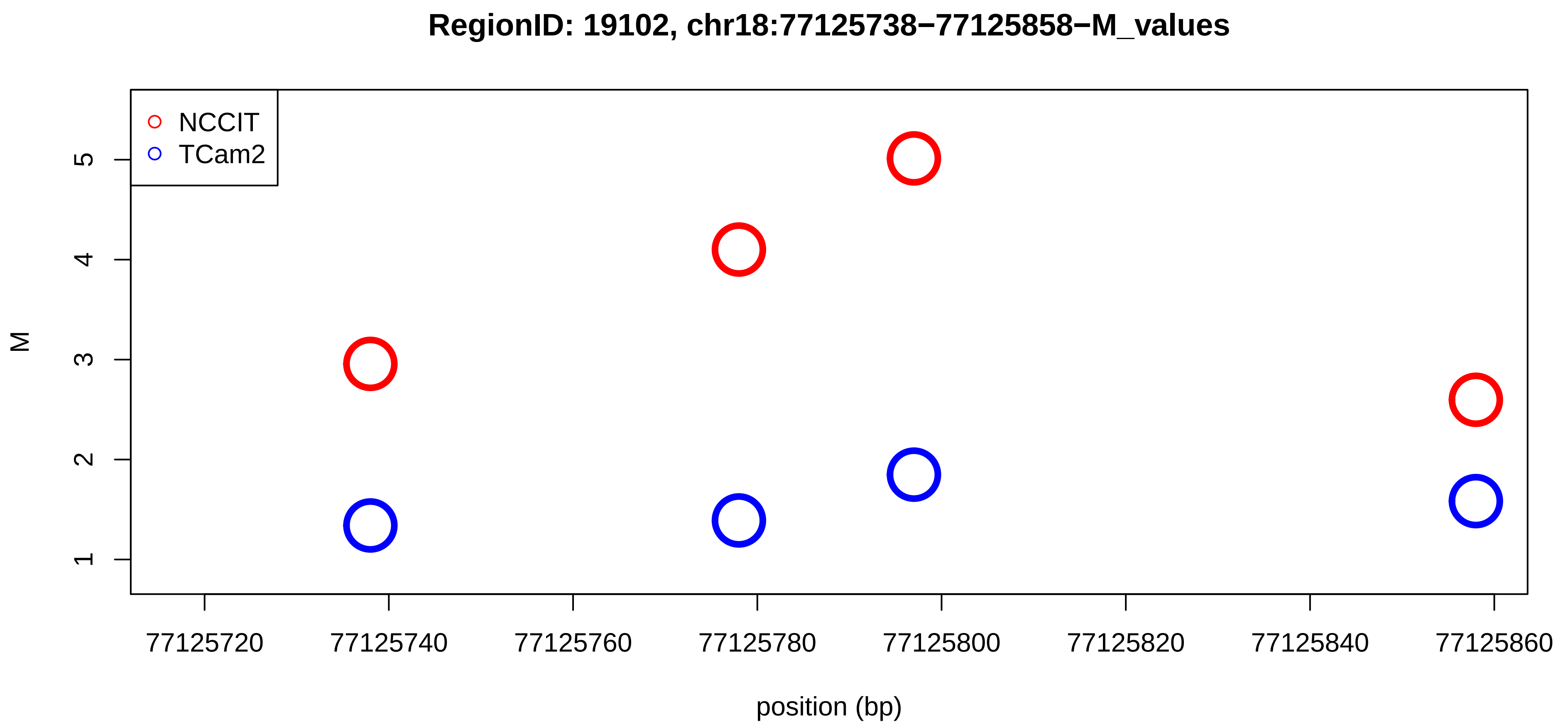
RegionID: 19102, chr18:77125738−77125858−M_values
NCCIT
TCam2
M
1
2
3
4
5
77125720
77125740
77125760
77125780
77125800
77125820
77125840
77125860
position (bp)

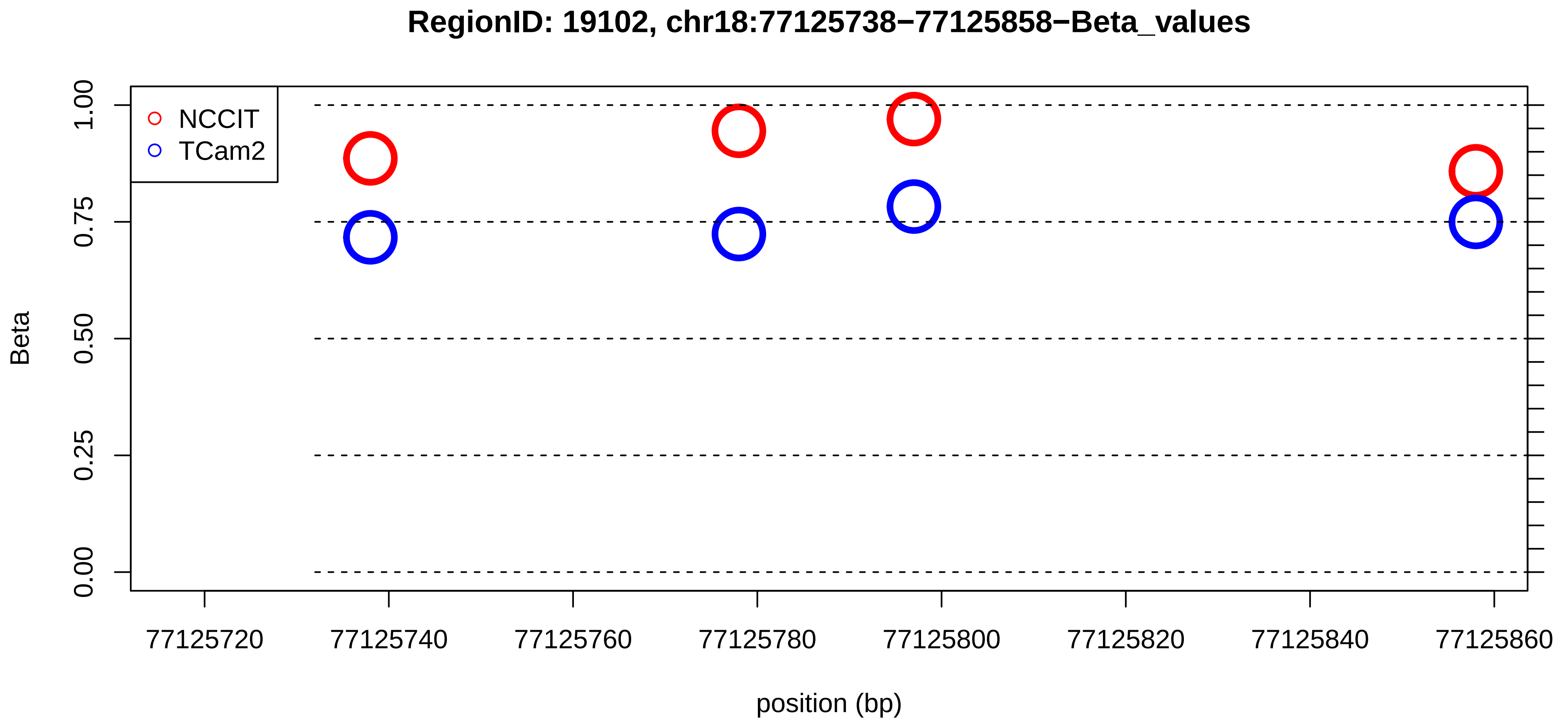
RegionID: 19102, chr18:77125738−77125858−Beta_values
NCCIT
TCam2
Beta
0.00
0.25
0.50
0.75
1.00
77125720
77125740
77125760
77125780
77125800
77125820
77125840
77125860
position (bp)
